# Tunable spinful matter wave valve

**DOI:** 10.1038/s41598-019-44218-y

**Published:** 2019-06-17

**Authors:** Yan-Jun Zhao, Dongyang Yu, Lin Zhuang, Xianlong Gao, Wu-Ming Liu

**Affiliations:** 1 Beijing National Laboratory for Condensed Matter Physics, Institute of Physics, Chinese Academy of Sciences, Beijing, 100190 China; 2 Faculty of Information Technology, School of Microelectronics, Beijing University of Technology, Beijing, 100124 People’s Republic of China; 3 School of Physical Sciences, University of Chinese Academy of Sciences, Beijing, 100190 China; 4 School of Physics, Sun Yat-Sen University, Guangzhou, 510275 China; 5 Department of Physics, Zhejiang Normal University, Jinhua, 321004 People’s Republic of China

**Keywords:** Atomic and molecular physics, Quantum physics

## Abstract

We investigate the transport problem that a spinful matter wave is incident on a strong localized spin-orbit-coupled Bose-Einstein condensate in optical lattices, where the localization is admitted by atom interaction only existing at one particular site, and the spin-orbit coupling arouse spatial rotation of the spin texture. We find that tuning the spin orientation of the localized Bose-Einstein condensate can lead to spin-nonreciprocal/spin-reciprocal transport, meaning the transport properties are dependent on/independent of the spin orientation of incident waves. In the former case, we obtain the conditions to achieve transparency, beam-splitting, and blockade of the incident wave with a given spin orientation, and furthermore the ones to perfectly isolate incident waves of different spin orientation, while in the latter, we obtain the condition to maximize the conversion of different spin states. The result may be useful to develop a novel spinful matter wave valve that integrates spin switcher, beam-splitter, isolator, and converter. The method can also be applied to other real systems, e.g., realizing perfect isolation of spin states in magnetism, which is otherwise rather difficult.

## Introduction

Ultracold atoms, where atom interaction and spin-orbit coupling (SOC) can be artificially synthesized, are an ideal platform for simulating many-body physics^1–4^. The wave-particle duality points out that particles can behave like waves and also vice versa^5^. Thus, it is of interest to investigate the matter wave properties of multiple cold atoms. Tunable via magnetic^6–9^ or optical^10,11^ Feshbach resonance, the atom interaction accounts for versatile intriguing phenomena featuring the transport of spinless matter waves^12–25^. Typically, a nonlinear impurity can blockade the transmission of a perturbative incident wave^20^. Besides, the discrete breather, resulted from nonlinear lattices, can be partially transmitted, and shifted by a moving breather^23^. Furthermore, when asymmetric defects are immersed in the nonlinear lattices, the discrete breather will be tilted, capably inducing the unidirectional transport of wave packets^25^. In spinor Bose-Einstein condensate (BEC), however, the interaction can be spin-dependent which induces the non-Abelian Josephson effect^26^.

Meanwhile, as a key ingredient for spin Hall effect^27,28^ and topological insulator^29–31^, SOC can be generated through non-Abelian gauge fields induced by the space variation of light^32–35^. In combination with atom interactions, SOC can affect the properties of localized modes or solitons in cold atom BEC^36–40^. For example, Rashba SOC and cubic attractive interactions together can give rise to two types of solitary-vortex complexes, respectively termed semivortices and mixed modes^36^. Using the parity and time reversal symmetries of a two-dimensional SOC BEC, localized solutions of various families, including multipole and half-vortex solitons, can be found^37^. Compact localized states and discrete solitons can coexist for nonlinear spinful waves on a flat-band network with SOC^40^. Although it has been reported^20^ that the localized BEC can blockade the propagation of an spinless incident wave, how to manipulate the transport of spinful matter waves via tunable nonlinearity in SOC BEC in optical lattices^41–49^ remains an open problem.

The research on matter wave manipulation is crucial to the recent advance of high-precision atomic-chip devices, such as coherent matter wave laser^50^, single-atom detector^51^, atomic clock and interferometer^52^, sensitive probes for acceleration, and rotation^53^, and detectors for tiny magnetic forces and gravity^53,54^. Generally, the gyroscope using matter wave Sagnac effect can exceed the conventional optical counterpart by almost ten orders of magnitude in terms of sensitivity. To realize more complicated high-precision detection in the future, one may require many atomic-chip devices to form a network. This furthermore need basic matter-wave-processing units analog to electromagnetic (microwave or optical) devices^55,56^, such as switcher, isolator, beam splitter, polarizer, etc. We will show that such analog devices for matter waves can be in principle achieved using SOC BEC with tunable nonlinearity.

In this paper, we investigate the transport problem that a weak transmission matter wave encounters a localized SOC BEC in optical lattices. In the presence of SOC, both the transmission and localized modes exhibit spin-rotation effect in the lattice space. The spin orientation, interaction and atom number of the BEC can be artificially manipulated, which induces tunable transport properties for incident waves with a definite spin orientation. In general, if the BEC orients parallel to the incident waves, it can behave like a spin switcher, beam-splitter, or isolator, while if they orient perpendicular, the BEC behaves like a spin converter.

Our paper is organized as follows. We introduce the mean-field approach to describe dynamics of the theoretical model. Using this approach, we afterwards focus on the transmission and local modes that are supported in this model. Then, we develop a perturbation method for treating the transpor of a weak matter wave that incomes on a strong localized BEC. Next, via specifying the concrete incident matter wave, we obtain the correponding scattering coefficients. After that, we discuss the transport properties based on the scattering coefficients. Lastly, the main results are discussed and concluded.

## Transmission and Localized Mode

### Theoretical model

**Figure 1:**
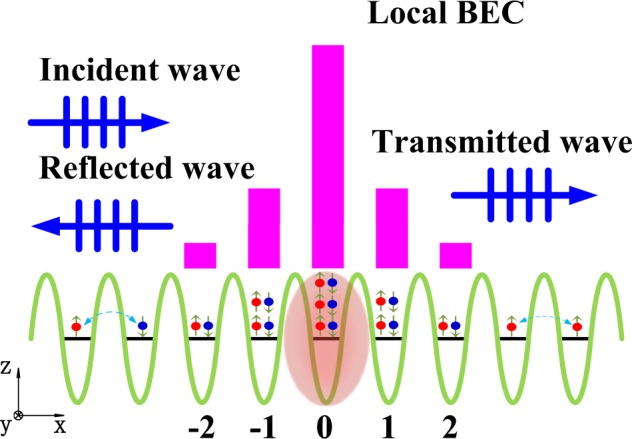
Scattering process of the weak atomic matter wave incident on the strong BEC localized in the vicinity of origin in optical lattices. Atoms are represented by red and blue balls with internal spins shown by arrows. The strong localized mode, whose magnitude is shown by magenta bars, is induced by localized interactions around origin (attractive, and theoretically idealized as a *δ*-type nonlinear impurity). The spin-flipping hopping between adjacent sites is aroused by SOC. The incident, reflected, and transmitted atoms with internal spins are represented as plane waves.

We consider the scattering process of the weak atomic matter wave incident on a spin-orbit coupled localized BEC in optical lattices (see Fig. 1). To create SOC, we can illuminate the ^87^Rb bosonic particles by two intersecting Raman lasers with proper magnetic bias, where the two internal atomic pseudo-spin-states are selected from within the ^87^Rb 5*S*_1/2_, *F* = 1 ground electronic manifold: |↑ = |*F* = 1, *m*_*F*_ = 0 (pseudo-spin-up) and |↓ = |*F* = 1, *m*_*F*_ = −1 (pseudo-spin-down)^57^. Besides, the optical lattices can be generated through a standing wave in the large detuning regime^14^. Moreover, the localization of the BEC can be induced by atom interaction concentrated on the vicinity of lattice origin, which can be obtained by generating inhomogeneous s-wave scattering length of atoms via tuning magnetic^6–9^ or optical^10,11^ Feshbach resonance.

In the second quantization form, the system can be well described by the Hamiltonian

1 $$\hat{H}=-\,J\sum _{n}\,({\hat{\psi }}_{n+1}^{\dagger }R{\hat{\psi }}_{n}+{\rm{H}}.{\rm{c}}.\,)-\sum _{n\sigma \sigma ^{\prime} }\,{\delta }_{n0}\frac{{U}_{\sigma \sigma ^{\prime} }}{2}{\hat{\psi }}_{n\sigma }^{\dagger }{\hat{\psi }}_{n\sigma ^{\prime} }^{\dagger }{\hat{\psi }}_{n\sigma ^{\prime} }{\hat{\psi }}_{n\sigma }.$$

where ${\hat{\psi }}_{n}={({\hat{\psi }}_{n\uparrow },{\hat{\psi }}_{n\downarrow })}^{\top }$ represents the macroscopic wave function of the BEC. The lattice potential well is deep enough to only involve the hopping between nearest neighbours. Concretely, the spin-conserving (spin-flipping) hopping is characterized by the diagonal (off-diagonal) terms of the spin-rotation operator *R* = exp(−*iσ*_*y*_*α*)^41,42^ which arises from the non-Abelian potential **A** = (*ασ*_*y*_, 0, 0) through Peierls substitution^58^. The SOC parameter *α* is determined by *α* = *πk*_soc_/*k*_ol_, where *k*_soc_ describes the momentum transfer from the Raman lasers and *k*_ol_ is the wave vector of the optical lattice^48,49^. By setting the intersection angle and wave length of the Raman lasers, we can tune *k*_soc_^57^ and furthermore, *α*. The localized attractive interaction is theoretically idealized as a *δ*-type nonlinear impurity, which vanishes except at *n* = 0. We choose the intraspecies interaction to fulfill *U*_↑↑_/*J* = *U*_↓↓_/*J* = *γ* and the interspecies interaction to fulfill *U*_↑↓_/*J* = *U*_↓↑_/*J* = *λγ*^41,42^ with *γ*, *λ* > 0 (attractive interaction). Hereafter, *γ* and *λ* are called interaction strength and miscibility parameter^59^, respectively.

To validate the mean-field approach, we hereafter assume *γ*, *λγ* ≪ 1, such that ${\hat{\psi }}_{n}$ and $\hat{H}$ can be mapped to the *c*-numbers *ψ*_*n*_, and *H*, respectively, i.e., ${\psi }_{n}={({\psi }_{n\uparrow },{\psi }_{n\downarrow })}^{\top }$ and

2 $$H=-\,J\sum _{n}\,({\psi }_{n+1}^{\dagger }R{\psi }_{n}+{\rm{H}}.{\rm{c}}.\,)-\sum _{n\sigma \sigma ^{\prime} }\,{\delta }_{n0}\frac{{U}_{\sigma \sigma ^{\prime} }}{2}{|{\psi }_{n\sigma }|}^{2}{|{\psi }_{n\sigma ^{\prime} }|}^{2}.$$

The dynamical evolution of the mean field *ψ*_*n*_ obeys the Gross-Pitaevskii equation, i.e., $i\partial {\psi }_{n\sigma }/\partial t=\partial H/\partial {\psi }_{n\sigma }^{\ast }$, yielding

3 $$i\frac{\partial {\psi }_{n}}{\partial t}=-\,R{\psi }_{n-1}-{R}^{\dagger }{\psi }_{n+1}-{\delta }_{n0}\gamma [\begin{array}{l}({|{\psi }_{n\uparrow }|}^{2}+\lambda {|{\psi }_{n\downarrow }|}^{2}){\psi }_{n\uparrow }\\ (\lambda {|{\psi }_{n\uparrow }|}^{2}+{|{\psi }_{n\downarrow }|}^{2}){\psi }_{n\downarrow }\end{array}].$$

Here, we have set the hopping strength *J* = 1 for simplicity.

We have stressed that the on-site attractive interaction only exists at origin, which will induce a localized mode where most atoms accumulate around *m* = 0 (see Fig. 1). Away from the nonlinear impurity, the matter wave can propagate freely along the optical lattices, which is called the transmission mode and governed only by the noninteracting terms in Eq. (1). Both modes can be solved using the Gross-Pitaevskii equation, which will be derived right below. Having obtained the solutions for both modes, we can furthermore seek the scattering coefficients for a weak transmission wave that is incident on the localized BEC mode.

### Transmission mode

We now seek the transmission modes using the Gross-Pitaevskii equation [Eq. (3)]. The transmission modes describe the matter wave that propagates freely along the optical lattices, which is governed by the noninteracting Hamiltonian of atoms [first term of Eq. (2)]. Accordingly, setting *γ* = 0 and *ψ*_*n*_ = *l*_*n*_exp(−*iωt*) in Eq. (3), the Gross-Pitaevskii equation, yields

4 $$\omega {l}_{n}=-\,R{l}_{n-1}-{R}^{\dagger }{l}_{n+1}.$$

Furthermore, we assume *l*_*n*_ has the form of spinful plane wave, i.e.,

5 $${l}_{n}={e}^{ink}{R}^{n}{l}_{0},$$

where *k* and *ω* are respectively the wave vector and eigenenergy of the transmission mode. In this assumption, Eq. (4) changes into

6 $$(\omega +2\,\cos \,k){l}_{0}=0,$$

which, because *l*_0_ should be nonzero spin states, gives the following dispersion relation

7 $$\omega =-\,2\,\cos \,k.$$

Obviously, the enregy *ω* is spin independent such that the solution of *l*_0_ is two-fold degenerate. The solution space of *l*_0_ can be any pair of spin states that own opposite spin orientations on the Bloch sphere. For example, corresponding to general opposite spin orientations

8 $${{\bf{s}}}_{\pm }=\pm \,({{\bf{e}}}_{x}\,\sin \,a\,\sin \,b+{{\bf{e}}}_{y}\,\cos \,a+{{\bf{e}}}_{z}\,\sin \,a\,\cos \,b),$$

where *a*, *b* ∈ [0, *π*] are azimuth and elevation angles, respectively. Such spin state pair can be represented as

9 $${l}_{+}=\,\cos (a/2){u}_{+}+{e}^{ib}\,\sin (a/2){u}_{-},$$

10 $${l}_{-}=-\,{e}^{-ib}\,\sin (a/2){u}_{+}+\,\cos (a/2){u}_{-}.$$

where ${u}_{\pm }={(1,\pm i)}^{\top }$ are eigenstates of *σ*_*y*_ *(σ*_*y*_*u*_±_ = ±*u*_±_). We can verify that the constraint *b* ∈ [0, *π*] (instead of *b* ∈ [0, 2*π*]) make**s s**_+ (_**s**_−_) always point to *x* > 0 (*x* < 0). One optional method to obtain *l*_±_ is namely solving the secular equation *σ*_0_*l*_±_ = ±*l*_±_. Here, the Pauli operator *σ*_0_ is obtained by calculating *σ*_0_ = **s**_+_⋅***σ***, which yields *σ*_0_ _=_ ***σ***_*x*_sin*a*sin*b* + *σ*_*y*_cos*a* +* σ*_*z*_sin*a*cos*b*.

Now we focus on the condition *l*_0_ = *l*_±_, where the transmission modes become

11 $${l}_{n}={l}_{\pm ,n}={e}^{ink}{R}^{n}{l}_{\pm }.$$

**Figure 2:**
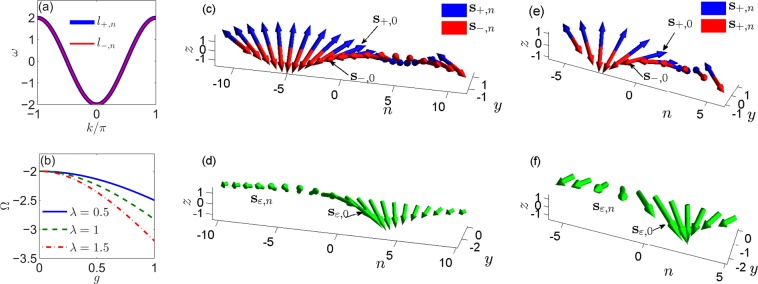
(**a**) Dispersion relation: energy *ω* against wave vector *k* for transmission modes *l*_+,*n*_ (solid blue) and *l*_−,*n*_ (solid red). (**b**) Eigenenergy Ω of the strong localized mode against localization grade *g* for miscibility parameter *λ* taking 0.5 (solid blue), 1 (dashed green), and 1.5 (dash-dotted red), respectively. (**c**,**e**) Spin texture of the transmission modes: ${{\bf{s}}}_{+,n}={l}_{+,n}^{\dagger }{\boldsymbol{\sigma }}{l}_{+,n}$ (blue) and ${{\bf{s}}}_{-,n}={l}_{-,n}^{\dagger }{\boldsymbol{\sigma }}{l}_{-,n}$ (red), where *a* = *π*/4 and *b* = *π*/2, thus **s**_±,0_ directing ± (***e***_*x*_ + ***e***_*y*_). (**d**,**f**) Spin texture of the localized mode: ${{\bf{s}}}_{\varepsilon ,n}={d}_{n}^{\dagger }{\boldsymbol{\sigma }}{d}_{n}$, where Ω = −2.01, making the spatial decay rate *κ* = 0.9049, and *ε* = *π*/4, thus **s**_*ε*,0_ directing **e**_*x*_ − **e**_*y*_. In (**c**–**f**), we see the rotation of spin orientation with *y*-axis for changing *n*, which is adjustable by the SOC parameter *α*. In (**c**,**d**), we assign *α* = *π*/20, such that the spin orientation recovers after the site changes by Δ*n* = *π*/*α* = 20, while, in (**e**,**f**), *α* = *π*/10 such that the recovery of spin orientation requires Δ*n* = *π*/*α* = 10.

In Fig. 2(a), we have plotted the dispersion relation for both *l*_±,*n*_. Apparently, the energy band of the transmission mode is −2 ≤ *ω* ≤ 2. Besides, one definite energy *ω* must result in four degenerate transmission modes:

12 $${l}_{n}^{(1,2)}={e}^{\pm ink}{R}^{n}{l}_{+},{l}_{n}^{(3,4)}={e}^{\pm ink}{R}^{n}{l}_{-},$$

where *k* = arccos(−*ω*/2) ∈ (0, *π*) is explicitly hypothesized. The spin orientations of *l*_±,*n*_ can be calculated by ${{\bf{s}}}_{\pm ,n}={l}_{\pm ,n}^{\dagger }{\boldsymbol{\sigma }}{l}_{\pm ,n}$, yielding

13 $${{\bf{s}}}_{\pm ,n}=\pm \,2[\,\sin \,a\,\sin (b+2na){{\bf{e}}}_{x}+\,\cos \,a{{\bf{e}}}_{y}+\,\sin \,a\,\cos (b+2na){{\bf{e}}}_{z}].$$

From Fig. 2(c), we can see that **s**_+,*n*_ and **s**_−,*n*_ always orient opposite. Impacted by the SOC parameter *α*, both **s**_±,*n*_ manifest spin-rotation effect with *y*-axis when the lattice site *n* changes. Apparently, the winding number per unit increment of the lattice site is *α*/*π*. The spin orientation will recover after the site changes by Δ*n* = *π*/*α*.

### Localized mode

We now solve the localized mode induced by the attractive atom interaction at origin. In contrast to the transmission modes, the localized mode is described by the full Hamiltonian *H* in Eq. (2), where *γ* is nonzero such that atom interactions are included. Accordingly, setting *γ* ≠ 0 and *ψ*_*n*_ = *d*_*n*_exp(−*i*Ω*t*) in Eq. (3), the Gross-Pitaevskii equation, yields

14 $${\rm{\Omega }}{d}_{n}=-\,R{d}_{n-1}-{R}^{\dagger }{d}_{n+1}-{\delta }_{n0}\gamma [\begin{array}{l}({|{d}_{n\uparrow }|}^{2}+\lambda {|{d}_{n\downarrow }|}^{2}){d}_{n\uparrow }\\ (\lambda {|{d}_{n\uparrow }|}^{2}+{|{d}_{n\downarrow }|}^{2}){d}_{n\downarrow }\end{array}].$$

Furthermore, we here assume the localized mode possesses the following profile

15 $${d}_{n}=\sqrt{\frac{g}{\gamma }}{\kappa }^{|n|}{R}^{n} {\mathcal E} ,$$

where |*κ*| < 1, and ${\mathcal E}$ is a two-component spin state. Inserting Eq. (15) into Eq. (14) for *n* = ±1, 0, we obtain the spatial decay rate *κ*, eigenenergy (or chemical potential) Ω, and spin state ${\mathcal E}$, i.e.,

16 $$\kappa =\frac{1}{2}(\,-\,{\rm{\Omega }}-\sqrt{{{\rm{\Omega }}}^{2}-4}),$$

17 $${\rm{\Omega }}=-\,\sqrt{{(1+\lambda )}^{2}{g}^{2}+4},$$

18 $$ {\mathcal E} ={({e}^{i\varepsilon },1)}^{\top }.$$

Here, *g* is called localization grade, which reflects the amplitude of the localized BEC and is a key factor to determine *κ* and Ω. The energy band of the localized mode is Ω < −2, as shown in Fig. 2(b), where we have plotted Ω against *g* for different *λ*. We see clearly decreasing of Ω with respect to the increase of *g* or *λ*.

Besides, the spin state ${\mathcal E} ={({e}^{i\varepsilon },1)}^{\top }$ mainly impacts the spin texture of *d*_*n*_, which is defined by ${{\bf{s}}}_{\varepsilon ,n}={d}_{n}^{\dagger }{\boldsymbol{\sigma }}{d}_{n}$, and can be further represented as

19 $${{\bf{s}}}_{\varepsilon ,n}=\frac{2g}{\gamma }{\kappa }^{2|n|}[{{\bf{e}}}_{x}\,\cos \,\varepsilon \,\cos (2n\alpha )-{{\bf{e}}}_{y}\,\sin \,\varepsilon -{{\bf{e}}}_{z}\,\cos \,\varepsilon \,\sin (2n\alpha )].$$

Similarly to **s**_±,*n*_, **s**_*ε*,*n*_ also manifests spin rotation effect due to the presence of SOC [see Fig. 2(d)], where the winding number per unit increment of the lattice site is also *α*/*π*. And the spin orientation will recover after the site changes by Δ*n* = *π*/*α*.

The atom number of the localized mode can be calculated by ${N}_{{\rm{at}}}=\sum _{n}\,{d}_{n}^{\dagger }{d}_{n}$, which yields ${N}_{{\rm{at}}}=\frac{-2{\rm{\Omega }}}{(1+\lambda )\gamma }.$ The localized mode only exists above a threshold: *N*_at_ > *N*_th_, given by *N*_th_ = 4/(1 + *λ*)*γ*. Also noting Ω is implicitly related to *λ* and *g* [see Eq. (17)], we can claim that the localization grade *g* is tunable via modifying the interaction strength *γ*, miscibility parameter *λ*, or atom number *N*_at_, i.e.,

20 $$g=\sqrt{\frac{{N}_{{\rm{at}}}^{2}{\gamma }^{2}}{4}-\frac{4}{{(\lambda +1)}^{2}}}.$$

Note that *γ*, *λ* (*N*_at_) can be controlled by Feshbach resonance (evaporative cooling) in typical cold atom experiment. Hence, the independent control of *λ* and *g* is feasible, which guarantees the tunability of our scheme.

## Solving the Transport Process

### Spinful plane wave interacting with the localized BEC

To investigate the transport process that a spinful plance wave encounters a localized BEC, we substitute *ψ*_*n*_ = *ϕ*_*n*_ + Ψ_*n*_ into the Gross-Pitaevskii equation [see Eq. (3)]. Here, Ψ_*n*_ = *d*_*n*_*e*^−*i*Ω*t*^, assumed strong, is the localized BEC while *ϕ*_*n*_, assumed weak, represents the incident and other stimulated waves. Rigorously, we assume $|{\varphi }_{0\sigma }|\ll |{{\rm{\Phi }}}_{0\sigma ^{\prime} }|=\sqrt{g/\gamma }$, thus resulting in the linearized Gross-Pitaevskii equation with respect to *ϕ*_*n*_:

21 $$i\frac{\partial {\varphi }_{n}}{\partial t}=-\,R{\varphi }_{n-1}-{R}^{\dagger }{\varphi }_{n+1}-{\delta }_{n0}({R}_{-}{\varphi }_{0}+R(t){R}_{+}{\varphi }_{0}^{\ast }).$$

One finds the strong localized BEC generates a non-Abelian potential at origin, which is quantified by the parameters *R*_±_ = *g*[*λ* + 2 + *λ*(cos*ε* ± *σ*_*y*_sin*ε*)] and *R*(*t*) = exp[*i*(*ε* − 2Ω*t* + *σ*_*z*_*ε*)]. Once encountered, the potential will scatter off a spinful plane wave or flip its spin, which will otherwise propagate freely governed only by the first two terms in Eq. (21).

We now justify the reasonability of our calculations in Eq. (21) taking into account the spatial profile of the localized BEC in Eq (15). Although there may be a few sites in the adjacent region of *n* = 0, where the atom numbers are larger than other sites, it does not affect our assumption that the interactions are only valid at *n* = 0. The reason is that the atom interactions are usually synthesized by the optical Feshbach resonance, which can be focused only on a single site *n* = 0, once a very thin lasing light beam is employed. Thus, even though the atom number may be large, lack of optical Feshbach resonance will still induce no interactions between atoms. The spatial profile of the localized BEC near *n* = 0 is fundamentally induced by the nearest hoppings between lattices, which does not contradict our assumption that the interaction is only valid at *n* = 0.

### Solving method

Now we discuss the method to solve the linearized Gross-Pitaevskii equation. Investigating the term $R(t){R}_{+}{\varphi }_{0}^{\ast }$ in Eq. (21), where *R*(*t*) is proportional to exp(−2Ω*t*), we are convinced to make the ansatz *ϕ*_*n*_ = *p*_*n*_*e*^−*iωt*^ + *q*_*n*_*e*^−*iνt*^ with *ν* = 2Ω − *ω*. Having done such a treatment, we can therefore obtain the coupled equations that feature the interplay between *p*_*n*_ and *q*_*n*_, i.e.,

22 $$\omega {p}_{n}=-\,R{p}_{n-1}-{R}^{\dagger }{p}_{n+1}-{\delta }_{n0}({R}_{-}{p}_{0}+{R}_{\varepsilon }{R}_{+}{q}_{0}^{\ast }),$$

23 $$\nu {q}_{n}=-\,R{q}_{n-1}-{R}^{\dagger }{q}_{n+1}-{\delta }_{n0}({R}_{-}{q}_{0}+{R}_{\varepsilon }{R}_{+}{p}_{0}^{\ast }),$$

with *R*_*ε*_ = exp[*i*(*ε* + *σ*_*z*_*ε*)]. Here, the symbol *ω* represents the energy of the incident wave which possess the wave vector *k*. Thus, the regime *ω* = −2cos*k* ∈ [−2, 2] means *p*_*n*_ is an extended state, which includes both the incident and scattered waves. Besides, noting that Ω, the energy of the localized BEC, is below −2, we can therefore obtain the energy *ν* < −2, a regime outside the energy band [−2, 2], which means *q*_*n*_ is a weak localized state stimulated by the incident wave.

To furthermore analyze the transport process, we need to specify the incident wave, which can be chosen from the transmission modes ${L}_{n}^{(j)}$ [see Eq. (12)] and differs in both the spin orientation and propagation direction. Since we have deliberately hypothesized 0 < *k* < *π* in Eq. (12), from the dispersion relation in Eq. (7), the group velocities *v*_*j*_ of the transmission mode ${l}_{n}^{(j)}$ must fulfill

24 $${v}_{1}={v}_{3}=2\,\sin \,k > 0,$$

25 $${v}_{2}={v}_{4}=-\,2\,\sin \,k < 0.$$

Thus, the incident waves coming from negative lattice sites should take the form

26 $${L}_{n}^{(j)}={l}_{n}^{(j)}{\theta }_{-n-1},j=1,3,$$

where *θ*_*n*_ is the Heaviside step function, i.e., *θ*_*n*_ = 1 if *n* ≥ 0 but *θ*_*n*_ = 0 otherwise, while the ones coming from positive should take

27 $${L}_{n}^{(j)}={l}_{n}^{(j)}{\theta }_{n},j=2,4.$$

In Eq. (28), the amplitude before ${L}_{n}^{(j)}$ has been set as unit, which does not influence the major physics since Eqs (22) and (23) are linear equations of *p*_*n*_ and *q*_*n*_. The spin orientations and propagation directions of all incident waves are summarized in Table 1.

**Table 1:** Propagation direction and spin orientation of the incident waves.

Incident wave	${l}_{n}^{(1)}$	${L}_{n}^{(2)}$	${l}_{n}^{(3)}$	${L}_{n}^{(4)}$
Propagation direction	→	←	→	←
Spin orientation	**s**_+_,*nθ* − *n* − 1	**s**_+_,*nθn*	**s**−,*nθ* − *n* − 1	*s*−,*nθn*

Here, *θ*_*n*_ is the Heaviside step function: *θ*_*n*_ = 1 if *n* ≥ 0 and *θ*_*n*_ = 0 if *n* < 0.

We now render the mathematical description of the transport process with respect to each incident wave ${L}_{n}^{(j)}$. In detail, we suppose *p*_*n*_ and *q*_*n*_ are respectively of the following forms,

28 $${p}_{n}^{(j)}={L}_{n}^{(j)}+({S}_{2j}{l}_{n}^{(2)}+{S}_{4j}{l}_{n}^{(4)}){\theta }_{-n-1}+({S}_{1j}{l}_{n}^{(1)}+{S}_{3j}{l}_{n}^{(3)}){\theta }_{n},$$

29 $${q}_{n}^{(j)}={R}^{n}{q}_{0}^{(j)}{\chi }^{|n|}.$$

Here, the parameter *S*_*j*′*j*_ is the scattering coefficient that measures the scattering intensity from the incident wave ${L}_{n}^{(j)}$ into the transmission mode ${l}_{n}^{(j^{\prime} )}$. To simplify the discussion, we can justify that the transport process is isotropic, e.g., *S*_12_ = *S*_21_ (see the section “Justification of the isotropy of the transport process” in the Supplementary Information). Therefore, only the cases of *j* = 1 and 3 merit detailed investigation, which means that we only focus on the incident waves coming from negative lattice sites.

### Scattering coefficients

Having determined the forms of *p*_*n*_ and *q*_*n*_, we can now access the final results. In detail, this can be achieved via inserting ${p}_{n}={p}_{n}^{(j)}$ [see Eq. (28)] and ${q}_{n}={q}_{n}^{(j)}$ [see Eq. (29)] into the coupled equations between them [see Eqs (22) and (23)] for *n* taking −1, 0, and 1, respectively.

After cancelling some variables, we can then obtain ${S}_{j^{\prime} j}$, the scattering coefficients, for *j* = 1, 3:

30 $${S}_{11}={S}_{21}+1=\frac{i\tilde{k}(i\tilde{k}+X+Y{C}_{Y})}{{(i\tilde{k}+X)}^{2}-{Y}^{2}},$$

31 $${S}_{31}={S}_{41}=\frac{i\tilde{k}(iY)(i{e}^{ib}\,\sin \,a\,\sin \,\varepsilon -{C}_{\varepsilon }\,\cos \,\varepsilon )}{{(i\tilde{k}+X)}^{2}-{Y}^{2}},s$$

32 $${S}_{33}={S}_{43}+1=\frac{i\tilde{k}(i\tilde{k}+X-Y{C}_{Y})}{{(i\tilde{k}+X)}^{2}-{Y}^{2}},$$

33 $${S}_{13}={S}_{23}=\frac{i\tilde{k}(iY)(i{e}^{-ib}\,\sin \,a\,\sin \,\varepsilon +{C}_{\varepsilon }^{\ast }\,\cos \,\varepsilon )}{{(i\tilde{k}+X)}^{2}-{Y}^{2}},$$

here, we have used the compact parameters $\tilde{k}=2{g}^{-1}\,\sin \,k$, *C*_*Y*_ = sin*ε*cos*a* − cos*ε*sin*a*sin*b*, and *C*_*ε*_ = cos^2^(*a*/2) + *e*^*i*2*b*^sin^2^(*a*/2). The expressions of *X* and *Y* are a bit cumbersome, which we thus give in the section “Intermediate parameters in the scattering coefficients” in the Supplementary Information. From Eqs (30–33), it is easy to get conscious that *S*_2*j*_ (*S*_4*j*_) can be deduced from *S*_1*j*_ (*S*_3*j*_), meaning that the reflected waves can be calcuated from the transmitted waves. On the other hand, we are only interested in the properties of the transmitted waves. Thus, besides *j* = 1, 3, there is only need to concentrate on *j*^′^ = 1, 3 for the scattering coefficients ${S}_{j^{\prime} j}$. The scattering coefficients *S*_11_ and *S*_33_ characterize the transmission intensity of the same transmission modes, thus also called transmission coefficients. However, *S*_13_ and *S*_31_ characterize the conversion between spin orienation**s s**_+,*n*_ and **s**_−,*n*_, thus also called conversion efficiencies.

Now we turn to the result of the weak localized state [see Eq. (29)], for which, we can obtain

34 $$\chi =\frac{1}{2}(\,-\,\nu -\sqrt{{\nu }^{2}-4}),$$

35 $${q}_{0}^{(j)}=-\,{({R}_{-}-\sqrt{{\nu }^{2}-4})}^{-1}{R}_{\varepsilon }{R}_{+}{p}_{0}^{(j)\ast }.$$

The validity of |*χ*| < 1 can be confirmed, agreeing with the localization feature of ${q}_{n}^{(j)}$. Besides, Eq. (35) bridges ${q}_{0}^{(j)}$ with ${p}_{0}^{(j)}$, which physically means the amplitude of ${q}_{n}^{(j)}$ is determined by the incident wave, considering that ${p}_{0}^{(j)}$ can be easily known from Eq. (28), where the amplitude before the incident wave ${L}_{n}^{(j)}$ has been set as unit.

## Transport Properties

### Discriminating spin-nonreciprocal and spin-reciprocal transport

Here, we will discuss the spin-nonreciprocal/spin-reciprocal transport, which, differently from the conventional nonreciprocal/reciprocal transport describing spatial unidirectional^25,60^/isotropic transport, means that the transport properties are dependent on/independent of the spin orientation of incident waves. We have stated that the transmission modes ${l}_{n}^{(1)}$ and ${l}_{n}^{(3)}$ have different spin orientations, i.e., **s**_+,*n*_ and **s**_−,*n*_ with **s**_+,*n*_ ≡ −**s**_−,*n*_ [see Fig. 2(c,e)]. To discriminate the spin-nonreciprocal and spin-reciprocal transport processes, we should compare (i) *S*_31_ with *S*_13_, and (ii) *S*_11_ with *S*_33_. However, we note that the identity |*S*_31_| ≡ |*S*_13_| is established, which means that appropriate adjustment of the global phases of ${l}_{n}^{(1)}$ or ${l}_{n}^{(3)}$ will lead to *S*_31_ ≡ *S*_13_. Therefore, the discrimination between the spin-nonreciprocal and spin-reciprocal transport can be merely done by comparing *S*_11_ with *S*_33_. We emphasize that the adjustment of global phase before ${l}_{n}^{(1)}$ or ${l}_{n}^{(3)}$ will not impact the values of *S*_11_ and *S*_33_.

We can find that the spin-nonreciprocal transport, or quantitatively, ${S}_{11}\equiv {S}_{33}$ (equivalent to $|{S}_{11}|\equiv |{S}_{33}|$), can be achieved when ${C}_{Y}\ne 0$, leading to

36 $$\tan \,\varepsilon \ne \,\tan \,a\,\sin \,b.$$

**Figure 3:**
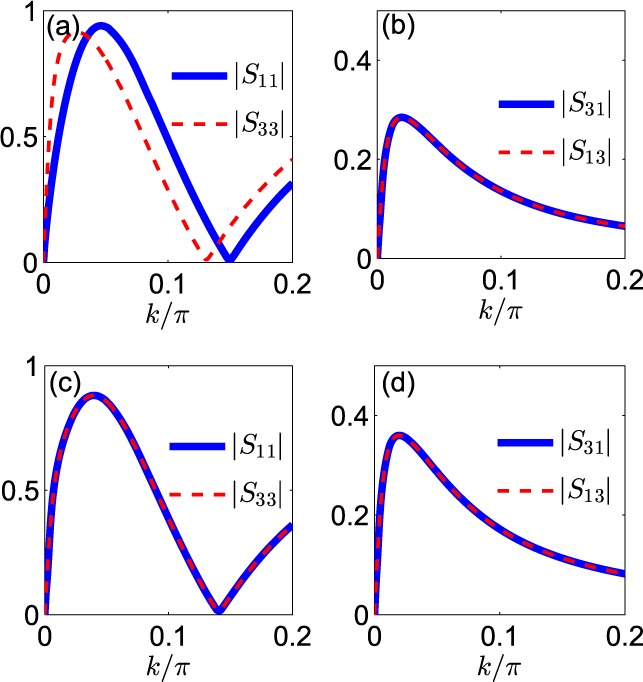
Modulis of the transmission coefficients (*S*_11_ and *S*_33_) and conversion coefficients (*S*_13_ and *S*_31_) plotted against *k*/*π* at (**a**,**b**) spin-nonreciprocal and (**c**,**d**) spin-reciprocal transport. In (**a**–**d**) we set the localization grade *g* = 0.69, miscibility parameter *λ* = 0.1, and angle parameters of the transmission modes *a* = *b* = *π*/4. However, in (**a**,**b**) to validate the spin-nonreciprocal transport condition *C*_*Y*_ ≠ 0, we specify *ε* = arctan(tan*a*tan*b*) − *π*/4 = 0, leading to *C*_*Y*_ = −0.6124. In (**c**,**d**) to validate the spin-reciprocal transport condition *C*_*Y*_ = 0, we specify the angle parameter of the localized BEC as *ε* = arctan(tan*a*sin*b*). In the cases of spin-nonreciprocal and spin-reciprocal transport, we respectively see $|{S}_{11}|\equiv |{S}_{33}|$ and |*S*_11_| ≡ |*S*_33_|, but |*S*_13_| ≡ |*S*_31_| holds in both cases.

In Fig. 3(a,b), we can see $|{S}_{11}|\equiv |{S}_{33}|$ and |*S*_13_| ≡ |*S*_31_| under the condition in Eq. (36). In contrast, the spin-reciprocal transport (*S*_11_ ≡ *S*_33_) can be achieved only when *C*_*Y*_ = 0, or rather,

37 $$\tan \,\varepsilon =\,\tan \,a\,\sin \,b.$$

In Fig. 3(c,d), we can see |*S*_11_| ≡ |*S*_33_| and |*S*_13_| ≡ |*S*_31_| under the condition in Eq. (37).

### Spin-Nonreciprocal Transport

#### Transparency, beam splitting, and blockade

Now we talk about the possibility of achieving transparency (*S*_*jj*_ = 1) and blockade (*S*_*jj*_ = 0) of the incident waves at spin-nonreciprocal transport. In the section “Transparency and blockade” in the Supplementary Information, we have demonstrated that both the transparency and blockade require *C*_*Y*_ = ∓1, that is, $b=\frac{\pi }{2}$ and $\varepsilon =a\mp \frac{\pi }{2}$. Here, $b=\frac{\pi }{2}$ means that **s**_±,0_ orient within the *xoy* plane [see Eq. (13)]. Meanwhile, $\varepsilon =a\mp \frac{\pi }{2}$ means that **s**_*ε*,*n*_ orients identical to **s**_±,*n*_, i.e., ${\tilde{{\bf{s}}}}_{\varepsilon ,n}\equiv {\tilde{{\bf{s}}}}_{\pm ,n}$, where

38 $${\tilde{{\bf{s}}}}_{c,n}=\frac{{{\bf{s}}}_{c,n}}{|{{\bf{s}}}_{c,n}|},(c=\varepsilon ,\pm )$$

is the normalized spin orientation. In detail, if ${\tilde{{\bf{s}}}}_{\varepsilon ,n}={\tilde{{\bf{s}}}}_{+,n}$, for the incident waves ${L}_{n}^{(1)}$ and ${L}_{n}^{(3)}$, the transparency will respectively occur at the points T1 and T2, which are defined by

39 $${\rm{T}}1:\mu -\frac{3}{2}(\lambda +1)=0,$$

40 $${\rm{T}}2:\mu +\frac{1}{2}(\lambda -3)(\lambda +1)=0,$$

with $\mu \equiv \mu (\omega )=\sqrt{{(\omega +2\sqrt{{(1+\lambda )}^{2}{g}^{2}+4})}^{2}-4}/g$; the blockade will respectively occur at B1 and B2, which are defined by

41 $${\rm{B}}1:\mu -2(\lambda +1)=0,$$

42 $${\rm{B}}2:\mu -2=0.$$

In contrast, if ${\tilde{{\bf{s}}}}_{\varepsilon ,n}={\tilde{{\bf{s}}}}_{-,n}$, for ${L}_{n}^{(1)}$ and ${L}_{n}^{(3)}$, the transparency points are respectively T2 and T1, and blockade points are respectively B2 and B1. The transparency and blockade points depending on the spin orientations have been summarized in Table 2. When the energy *ω* deviates from the transparency and blockade points, the incident waves will manifest partial transmission, which can be interpreted as the beam-splitting effect. We emphasize that *C*_*Y*_ = ∓1 leads to *S*_31_ = *S*_13_ = 0, signifying no conversion between ${l}_{n}^{(1)}$ and ${l}_{n}^{(3)}$ in the output fields.

**Table 2:** Transparency point (T. P.) and blockade point (B. P.) dependent on the spin orientations.

Incident wave	${\tilde{{\bf{s}}}}_{\varepsilon ,n}={\tilde{{\bf{s}}}}_{+,n}$		${\tilde{{\bf{s}}}}_{\varepsilon ,n}={\tilde{{\bf{s}}}}_{-,n}$		${\tilde{{\bf{s}}}}_{\varepsilon ,n}\ne {\tilde{{\bf{s}}}}_{\pm ,n}$	
	T. P.	B. P.	T. P.	B. P.	T. P.	B. P.
${L}_{n}^{(1)}$	T1	B1	T2	B2	None	None
${L}_{n}^{(3)}$	T2	B2	T1	B1	None	None

Here, *s*_*ε*,*n*_ is the normalized spin orientation of the localized BEC, while **s**_+,*n*_ (*s*_−,*n*_) is that of the incident wave ${L}_{n}^{(1)}$ (${L}_{n}^{(3)}$). The points T1, T2, B1, and B2 are given by Eqs (39–42). Note that *g* is the localization grade and *λ* is the miscibility parameter. Besides, Ω (*ω*) is the energy of the localized BEC (incident wave).

**Figure 4:**
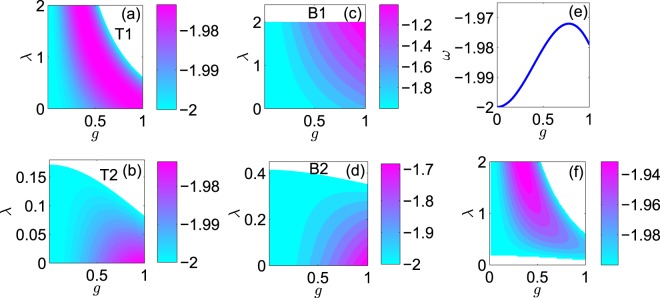
Energy *ω* against miscibility parameter *λ* and localization grade *g* at transparency points (**a**) T1, (**b**) T2, and blockade points (**c**) B1, (**d**) B2. (**e**) Energy *ω* against *g* at the isolation point. (**f**) Energy *ω* plotted against *g* and *λ* at the maximum spin conversion point. In (**a**–**d**,**f**), we only concentrate on the interesting parameter regime 0 ≤ *g* ≤ 1 and 0 ≤ *λ* ≤ 2, within which the white region signifies no solution of *ω* is found.

In Fig. 4(a–d), we have shown the controllability of the transparency and blockade points with tunable parameters *g* and *λ*. We find that, at T1, the larger energy *ω* appears in a ribbon-like region near the right upper boundary. At T2, the larger *ω* appears at larger *g* but smaller *λ*. At B1 and B2, the larger *ω* appears at both larger *g* and larger *λ*.

**Figure 5:**
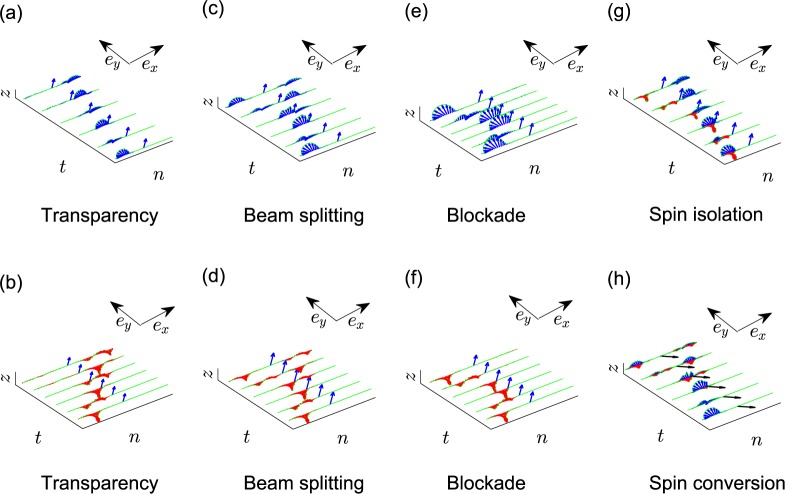
Time-evolution simulation of the transport processes: (**a**,**b**) transparency, (**c**,**d**) beam splitting, (**e**,**f**) blockade, (**g**) spin isolation, and (**h**) spin conversion. The blue (red) arrows mean spin orientation along ${\tilde{{\bf{s}}}}_{+,n}$ (${\tilde{{\bf{s}}}}_{-,n}$). In (**a**–**g**), we specify $b=\frac{\pi }{2}$, $a=\frac{\pi }{4}$, and $\varepsilon =a-\frac{\pi }{2}$ such that ${\tilde{{\bf{s}}}}_{\varepsilon ,n}={\tilde{{\bf{s}}}}_{+,n}=-\,{\tilde{{\bf{s}}}}_{-,n}$, while, in (**h**), *b* and *a* remains the same except *ε* = *a*, which makes ${\tilde{{\bf{s}}}}_{\varepsilon }\cdot {\tilde{{\bf{s}}}}_{\pm ,n}=0$. Transparency: the energy *ω* of the incident wave taken at (**a**) T1 and (**b**) T2 with localization grade *g* = 0.9 and miscibility parameter *λ* = 0.025. Beam splitting: (**c**) the energy *ω* = −1.9263 and (**d**) *ω* = −1.9670 with *g* = 0.69 and *λ* = 0.1. Blockade: *ω* taken at (**e**) B1 and (**f**) B2 with *g* = 0.75 and *λ* = 0.1. (**g**) Spin isolation: *ω* taken according to Eq. (44) with *g* = 0.7788 and $\lambda =\frac{1}{3}$. (**h**) Spin conversion: *ω* taken according to Eq. (46) with *g* = 0.5 and *λ* = 1. In (**a**,**c**), (**e**,**h**) [(**b**,**d**,**f**)], the incident wave is initialized with Eq. (43) for *j* taking 1 (*j* taking 3), while in (**g**), it is initialized according to Eq. (45).

In Fig. 5(a–f), we present the simulation result using the exact Gross-Pitaevskii equation [see Eq. (3)], where, for the incident wave ${L}_{n}^{(j)}$ (*j* = 1, 3), the perturbative part is initialized with a Gaussian profile:

43 $${\varphi }_{n}(0)={s}_{0}\exp [\,-\,{s}_{p}{(n-{n}_{0})}^{2}]{L}_{n}^{(j)}.$$

The angle parameters are specified as $b=\frac{\pi }{2}$, $a=\frac{\pi }{4}$, and $\varepsilon =a-\frac{\pi }{2}$ such that **s**_*ε*_ is identical (opposite) to **s**_+,*n*_ (**s**−_,*n*_): ${\tilde{{\bf{s}}}}_{\varepsilon ,n}\equiv {\tilde{{\bf{s}}}}_{+,n}\equiv -\,{\tilde{{\bf{s}}}}_{-,n}$. Figure 5(a,b) respectively show the transparency at T1 and T2 for the incident waves ${L}_{n}^{(1)}$ and ${L}_{n}^{(3)}$. Figure 5(c,d) respectively show the beam splitting effect for the incident waves ${L}_{n}^{(1)}$ and ${L}_{n}^{(3)}$. Figure 5(e,f) respectively show the blockade at B1 and B2 for the incident waves ${L}_{n}^{(1)}$ and ${L}_{n}^{(3)}$. Thus, we can claim that tunable transport is achievable from transparency, beam splitting, to blockade.

#### Spin isolation

Now, under the condition ${\tilde{{\bf{s}}}}_{\varepsilon ,n}={\tilde{{\bf{s}}}}_{\pm ,n}$, we continue to explore the possibility of achieving perfect isolation of different spin states, that is, making one spin state fully transmitted and the other totally reflected. To this end, there are two possible situations: (i) T1 and B2 overlaps, yielding $\lambda =\frac{1}{3}$; (ii) T2 and B1 overlaps, yielding *λ* = −1. The case *λ* = −1 exceeds the scope of the present discussion. Therefore, we only concentrate on $\lambda =\frac{1}{3}$, with energy *ω* determined by

44 $$\mu (\omega )=2.$$

**Figure 6:**
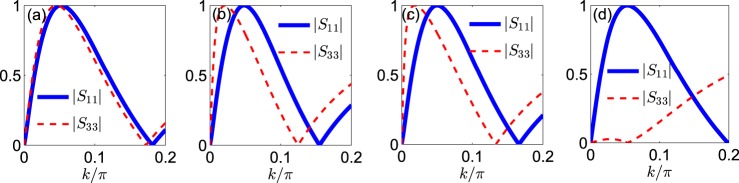
Modulis of transmission coefficients *S*_11_ and *S*_33_ at spin-nonreciprocal transport: (**a**) *g* = 0.9, *λ* = 0.025; (**b**) *g* = 0.69, *λ* = 0.1; (**c**) *g* = 0.75, *λ* = 0.1; (**d**) *g* = 0.7788, $\lambda =\frac{1}{3}$. Besides, *ε* = *a* − *π*/2, which makes ${\tilde{{\bf{s}}}}_{\varepsilon ,n}\equiv {\tilde{{\bf{s}}}}_{+,n}$.

As an example, we specify $\varepsilon =a-\frac{\pi }{2}$ to make ${\tilde{{\bf{s}}}}_{\varepsilon ,n}\equiv {\tilde{{\bf{s}}}}_{+,n}$, in which case, the transparency and blockade points of ${L}_{n}^{(1)}$ [${L}_{n}^{(3)}$] are respectively T1 (T2) and B1 (B2). The perfect isolation of spin states, i.e., |*S*_11_| = 1 and |*S*_33_| = 0, can not be achieved simultaneously [see Fig. 6(a–c)], unless $\lambda =\frac{1}{3}$ [see Fig. 6(d)]. Similarly, if we specify $\varepsilon =a+\frac{\pi }{2}$, which makes ${\tilde{{\bf{s}}}}_{\varepsilon ,n}\equiv {\tilde{{\bf{s}}}}_{-,n}$, |*S*_11_| = 0 and |*S*_33_| = 1 are then achievable simultaneously if $\lambda =\frac{1}{3}$. The controllability of the isolation point determined by Eq. (44) is shown in Fig. 4(e). In Fig. 5(g), we present the simulation result using the exact Gross-Pitaevskii equation [see Eq. (3)], where the perturbative part is initialized with a Gaussian profile

45 $${\varphi }_{n}(0)={s}_{0}\exp [\,-\,{s}_{p}{(n-{n}_{0})}^{2}]({L}_{n}^{(1)}+{L}_{n}^{(3)}).$$

As in Fig. 5, we still specify $b=\frac{\pi }{2}$, $a=\frac{\pi }{4}$, and $\varepsilon =a-\frac{\pi }{2}$ to make ${\tilde{{\bf{s}}}}_{\varepsilon ,n}={\tilde{{\bf{s}}}}_{+,n}=-\,{\tilde{{\bf{s}}}}_{-,n}$.

### Spin-reciprocal transport: spin conversion

We are also curious about the conversion between the spinful waves ${l}_{n}^{(1)}$ and ${l}_{n}^{(3)}$. The conversion efficiencies are namely the scattering coefficiencies *S*_31_ and *S*_13_. To maximize the conversion efficiencies, the condition tan*ε* = tan*a*sin*b* should be satisfied, resulting in *C*_*Y*_ = 0 (see the section “Spin conversion” in the Supplementary Information). In contrast to the transparency and blockade cases, this means **s**_*ε*_ must orient perpendicular to **s**_±,*n*_, i.e., ${\tilde{{\bf{s}}}}_{\varepsilon }\cdot {\tilde{{\bf{s}}}}_{\pm ,n}=0$. Meanwhile, the relation *S*_11_ = *S*_33_ is caused, implying spin-reciprocal transport behaviours which are independent of the spin orientation of incident waves. Moreover, the energy *ω* of the incident wave is required to satisfy

46 $$4-{\omega }^{2}={g}^{2}({Y}^{2}-{X}^{2}),$$

which is called maximum spin conversion point [see Fig. 4(f)], where $$X=(2+\lambda )+\frac{({\lambda }^{2}+1)\mu -{\lambda }^{3}-\lambda -2}{(\mu -2)(\mu -2-2\lambda )},Y=$$$$\lambda [1+\frac{2\mu +{\lambda }^{2}-2\lambda -3}{(\mu -2)(\mu -2-2\lambda )}]$$. Under these conditions, the maximum conversion efficiency can be achieved as $$|{S}_{31}|=|{S}_{13}|=\frac{1}{2}$$. In Fig. 5(h), we present the simulation result using the exact Gross-Pitaevskii equation [see Eq. (3)], where the perturbative part is initialized with a Gaussian profile: $${\varphi }_{n}(0)={s}_{0}\exp [\,-\,{s}_{p}{(n-{n}_{0})}^{2}]{L}_{n}^{(1)}$$. Besides, we specify $$b=\frac{\pi }{2}$$, $$a=\frac{\pi }{4}$$, and *ε* = *a* such that $${\tilde{{\bf{s}}}}_{\varepsilon ,n}\cdot {\tilde{{\bf{s}}}}_{\pm ,n}=0$$.

## Discussion and Conclusion

In experiment, the incident ^87^Rb atoms can acquire the quasimomentum *k* via phase imprinting method (i.e., using an off-resonant light pulse to generate a proper light-shift potential which dominates the evolution of the initial BEC wavepacket)^61^, Bragg scattering, or simply acceleration of the matter-wave probe in an external potential. The spin of the BEC can be manipulated by Rabi oscillation induced by Raman laser pulses that couple internal spin states with two-photon resonance. To measure the scattering atoms, we first use a Stern–Gerlach gradient to separate atoms of different spin states whose quantity can be further calculated via absorption imaging^57^.

In conclusion, we have investigated the transport of a spinful matter wave scattered by a strong localized BEC, in which the matter wave undergoes spin rotation along optical lattices due to the presence of SOC, and the strong localized BEC generates an effective non-Abelian potential to the spinful wave which furthermore impacts its transport behaviour. Tuning the spin of the localized BEC to orient parallel to that of the incident wave, we can achieve transparency, blockade, and beam splitting of the incident wave. However, both the transparency and blockade points are different for two incident waves with opposite spin orientation. Thus, it is feasible to isolate two waves of different spin orientation. In contrast, the maximum conversion between matter waves with opposite spin orientation can also be achieved once the localized BEC is tuned to orient perpendicular to the incident waves. The conditions to realize different transport properties are summarized in Table 3.

**Table 3:** Summary of the transport properties and the corresponding conditions.

Transport properties	Spin direction requirement	Interaction requirement	Energy requirement
Blockade	$b=\frac{\pi }{2},\varepsilon =a\mp \frac{\pi }{2}$		*μ* − 2(*λ* + 1) = 0 or *μ* − 2 = 0
Transparency	$b=\frac{\pi }{2},\varepsilon =a\mp \frac{\pi }{2}$		$\mu -\frac{3}{2}(\lambda +1)=0$ or $\mu +\frac{1}{2}(\lambda -3)(\lambda +1)=0$
Spin isolation	$b=\frac{\pi }{2},\varepsilon =a\mp \frac{\pi }{2}$	$\lambda =\frac{1}{3}$	*μ* = 2
Spin conversion	tan*ε* = tan*a*sin*b*		4 − *ω*^2^ = *g*^2^(*Y*^2^ − *X*^2^)

The “Spin direction requirement” column gives the parameter requirement for the relative spin direction, where the angel(s) *ε* (*a*, *b*) characterizes the spin direction of the BEC (incident plane wave). Besides, $\mu \equiv \mu (\omega )=\sqrt{{(\omega +2\sqrt{{(1+\lambda )}^{2}{g}^{2}+4})}^{2}-4}/g$. Here, the localization grade *g* characterizes the amplitude of the BEC [see Eq. (15)] and can be tunable via the BEC atom number *N*_at_ or the interaction strength *γ*: $g=\sqrt{{N}_{{\rm{at}}}^{2}{\gamma }^{2}/4-4/{(\lambda +1)}^{2}}$. The parameter *γ* is the interaction strength*, λ* is the miscibility parameter, and *ω* is the energy of the incident wave. The compact expressions of *X* and *Y* are $X=(2+\lambda )+\frac{({\lambda }^{2}+1)\mu -{\lambda }^{3}-\lambda -2}{(\mu -2)(\mu -2-2\lambda )},Y=\lambda [1+\frac{2\mu +{\lambda }^{2}-2\lambda -3}{(\mu -2)(\mu -2-2\lambda )}]$.

The result may be heuristic for developing a novel spinful matter wave valve that integrates spin switcher, beam splitter, isolator, and converter on a single atomic chip. As basic matter-wave-processing units similar to microwave^55^ or optical^56^ switcher, beam splitter, isolator, and polarizer, such valves may help to form more complicated network formed by high-precision atomic-chip devices. The proposal extends the atomtronics^62^ to a spinful case, i.e., a matter-wave version of spintronics, which is believed to give insights in many quantum-based applications such as gravitometry, magnetometry, etc. Also, our proposal may facilitate the perfect isolation of spin states in magnetism, which is otherwise rather difficult.

## Supplementary information

Supplementary information for “Tunable spinful matter wave valve”
