# Supplementary material for: Tunable spinful matter wave valve
Source: Sci Rep. 2019 Jun 17;9:8653. doi: 10.1038/s41598-019-44218-y (PMC6572844; doi:10.1038/s41598-019-44218-y)
Supplement: Supplementary file 1 — Supplementary information for “Tunable spinful matter wave valve” [file 41598_2019_44218_MOESM1_ESM.pdf]

Supplementary information for “Tunable spinful matter wave valve”

Yan-Jun Zhao[1,2,3], Dongyang Yu[1,3], Lin Zhuang[4], and Wu-Ming Liu[1,3,*]

[1]Beijing National Laboratory for Condensed Matter Physics, Institute of Physics, Chinese Academy of Sciences, Beijing 100190, China
[2]Faculty of Information Technology, School of Microelectronics, Beijing University of Technology, Beijing 100124, People’s Republic of China
[3]School of Physical Sciences, University of Chinese Academy of Sciences, Beijing 100190, China
[4]School of Physics, Sun Yat-Sen University, Guangzhou 510275, China
[*]wliu@iphy.ac.cn

ABSTRACT

This document provides supplementary information to some important results presented in the main text.

Justification of the isotropy of the transport process

We now justify the transport process must be isotropic. Suppose the solution is $p_n = p_n^{(j)} \equiv p_n^{(j)}(\alpha)$ and $q_n = q_n \equiv q_n^{(j)}(\alpha)$, then we can obtain the coupled equations for the solution:

$$\begin{aligned}\omega p_n^{(j)}(\alpha) =& -R(\alpha)\, p_{n-1}^{(j)}(\alpha) - R^{\dagger}(\alpha)\, p_{n+1}^{(j)}(\alpha)\\ &-\delta_{n0}\left[R_- p_0^{(j)}(\alpha) + R_\varepsilon R_+ q_0^{(j)*}(\alpha)\right], \qquad (1)\\ \nu q_n^{(j)}(\alpha) =& -R(\alpha)\, q_{n-1}^{(j)}(\alpha) - R^{\dagger}(\alpha)\, q_{n+1}^{(j)}(\alpha)\\ &-\delta_{n0}\left[R_- q_0^{(j)}(\alpha) + R_\varepsilon R_+ p_0^{(j)*}(\alpha)\right]. \qquad (2)\end{aligned}$$

To remove the dependence of α, we now let $\tilde{p}_n^{(j)} = [R^n(\alpha)]^{\dagger}\, p_n^{(j)}(\alpha)$ and $\tilde{q}_n^{(j)} = [R^n(\alpha)]^{\dagger}\, q_n^{(j)}(\alpha)$, which leads to

$$\omega \tilde{p}_n^{(j)} = -\tilde{p}_{n-1}^{(j)} - \tilde{p}_{n+1}^{(j)} - \delta_{n0}\left[R_- \tilde{p}_0^{(j)} + R_\varepsilon R_+ \tilde{q}_0^{(j)*}\right], \qquad (3)$$

$$\nu \tilde{q}_n^{(j)} = -\tilde{q}_{n-1}^{(j)} - \tilde{q}_{n+1}^{(j)} - \delta_{n0}\left[R_- \tilde{q}_0^{(j)} + R_\varepsilon R_+ \tilde{p}_0^{(j)*}\right]. \qquad (4)$$

In the above, replacing n with $-n$, we arrive at

$$\begin{aligned}\omega \tilde{p}_{-n}^{(j)} =& -\tilde{p}_{-(n+1)}^{(j)} - \tilde{p}_{-(n-1)}^{(j)} - \delta_{n0}\left[R_- \tilde{p}_0^{(j)} + R_\varepsilon R_+ \tilde{q}_0^{(j)*}\right],\\ \nu \tilde{q}_{-n}^{(j)} =& -\tilde{q}_{-(n+1)}^{(j)} - \tilde{q}_{-(n-1)}^{(j)} - \delta_{n0}\left[R_- \tilde{q}_0^{(j)} + R_\varepsilon R_+ \tilde{p}_0^{(j)*}\right]. \qquad (5)\end{aligned}$$

Noting $R^n(\alpha) = \exp(-i\sigma_y n\alpha) = R^{-n}(-\alpha)$, we can obtain $p_{-n}^{(j)}(-\alpha) = R^n(\alpha)\, \tilde{p}_{-n}^{(j)}$ and $q_{-n}^{(j)}(-\alpha) = R^n(\alpha)\, \tilde{q}_{-n}^{(j)}$. Then, multiplying Eq. (5) with $R^n(\alpha)$, we find

$$\begin{aligned}\omega p_{-n}^{(j)}(-\alpha) =& -p_{-(n+1)}^{(j)}(-\alpha) - p_{-(n-1)}^{(j)}(-\alpha)\\ &-\delta_{n0}\left[R_- \tilde{p}_0^{(j)} + R_\varepsilon R_+ \tilde{q}_0^{(j)*}\right], \qquad (6)\\ \nu q_{-n}^{(j)}(-\alpha) =& -q_{-(n+1)}^{(j)}(-\alpha) - q_{-(n-1)}^{(j)}(-\alpha)\\ &-\delta_{n0}\left[R_- \tilde{q}_0^{(j)} + R_\varepsilon R_+ \tilde{p}_0^{(j)*}\right], \qquad (7)\end{aligned}$$

which means $p_n = p_{-n}^{(j)}(-\alpha)$ and $q_n = q_{-n}^{(j)}(-\alpha)$ also constitute the solution of the coupled equation. This solution is equivalent to $p_n = \mathscr{P}_{12}\mathscr{P}_{34}p_n^{(j)}(\alpha)$ and $q_n = \mathscr{P}_{12}\mathscr{P}_{34}q_n^{(j)}(\alpha)$, since they share the same incident wave. Here, $\mathscr{P}_{j_1 j_2}$ designates the permutation operator acting on indices j, e.g., $\mathscr{P}_{12}\mathscr{P}_{34}p_n^{(1)}(\alpha) = p_n^{(2)}(\alpha)$ and $\mathscr{P}_{12}\mathscr{P}_{34}p_n^{(3)}(\alpha) = p_n^{(4)}(\alpha)$. By comparing the terms between $p_{-n}^{(j)}(-\alpha)$ and $\mathscr{P}_{12}\mathscr{P}_{34}p_n^{(j)}(\alpha)$, we obtain the identity $S_{j'j} = \mathscr{P}_{12}\mathscr{P}_{34}S_{j'j}$, e.g., $S_{12} = S_{21}$, a straightforward proof of isotropic transport. For example, by specifying $j = 1$, we have

$$\begin{aligned} p_{-n}^{(1)}(-\alpha) =& l_n^{(2)}\theta_{n-1} + \left[S_{21}l_n^{(1)} + S_{41}l_n^{(3)}\right]\theta_{n-1} \\ &+ \left[S_{11}l_n^{(2)} + S_{31}l_n^{(4)}\right]\theta_{-n}, \end{aligned} \tag{8}$$

and

$$\begin{aligned} \mathscr{P}_{12}\mathscr{P}_{34}p_n^{(1)}(\alpha) =& p_n^{(2)}(\alpha) \\ =& l_n^{(2)}\theta_n + \left[S_{12}l_n^{(1)} + S_{32}l_n^{(3)}\right]\theta_n \\ &+ \left[S_{22}l_n^{(2)} + S_{42}l_n^{(4)}\right]\theta_{-n-1}. \end{aligned} \tag{9}$$

The relation $p_{-n}^{(1)}(-\alpha) \equiv \mathscr{P}_{12}\mathscr{P}_{34}p_n^{(j)}(\alpha)$ can naturally yield $S_{12} = S_{21}$, $S_{31} = S_{42}$. To this end, we are convinced to only investigate the scenarios with incoming waves from negative lattice sites, i.e., $L_n^{(j)}$ with $j = 1,3$.

Intermediate parameters in the scattering coefficients

In the expressions of the scattering coefficients S_{11}, S_{33}, S_{31}, and S_{13}, we have used the intermediate parameters X and Y, which take the following forms,

$$X = (2+\lambda) + \frac{(\lambda^2+1)\mu - \lambda^3 - \lambda - 2}{(\mu-2)(\mu-2-2\lambda)}, \tag{10}$$

$$Y = \lambda\left[1 + \frac{2\mu + \lambda^2 - 2\lambda - 3}{(\mu-2)(\mu-2-2\lambda)}\right], \tag{11}$$

where $\mu = g^{-1}\sqrt{(2\Omega-\omega)^2 - 4} = \sqrt{(\omega + 2\sqrt{(1+\lambda)^2g^2+4})^2 - 4}/g$.

Phenomena in the transport process

Transparency and blockade

We now explore the conditions for transparency of the incident wave. In this case of $j = 1$, the transmission coefficients for the incident wave is

$$\begin{aligned} S_{11} =& \frac{i\tilde{k}\left(i\tilde{k} + X + YC_Y\right)}{\left(i\tilde{k} + X\right)^2 - Y^2} \\ =& \frac{i\tilde{k}\left(i\tilde{k} + X + YC_Y\right)}{\left(i\tilde{k} + X + Y\right)\left(i\tilde{k} + X - Y\right)}. \end{aligned} \tag{12}$$

On the other hand, we note $C_Y = \sin\varepsilon\cos a - \cos\varepsilon\sin a\sin b = \sqrt{\cos^2 a + \sin^2 a \sin^2 b}Y\sin(\varepsilon - c) \in [-1,1]$, where $c = \arctan(\sin b\tan a)$. Thus, we furthermore have $|X + YC_Y| \leq |X+Y|$ or $|X+YC_Y| \leq |X-Y|$, which leads to

$$\begin{aligned} |S_{11}| =& \left|\frac{i\tilde{k}}{\left(i\tilde{k}+X+Y\right)}\right|\left|\frac{i\tilde{k}+X+YC_Y}{i\tilde{k}+X-Y}\right| \\ =& \left|\frac{i\tilde{k}+X+YC_Y}{\left(i\tilde{k}+X+Y\right)}\right|\left|\frac{i\tilde{k}}{i\tilde{k}+X-Y}\right| \leq 1. \end{aligned} \tag{13}$$

Here, the "=" sign is achieved when

$$C_Y = \mp 1, X \pm Y = 0.$$

In this condition, we actually have $S_{11}=1$, meaning the incident wave $L_n^{(1)}$ is transparent. In the case of $j=3$, the transmission coefficients for the incident wave is

$$S_{33}=\frac{i\tilde{k}\left(i\tilde{k}+X-YC_Y\right)}{\left(i\tilde{k}+X\right)^2-Y^2}=\frac{i\tilde{k}\left(i\tilde{k}+X-YC_Y\right)}{\left(i\tilde{k}+X+Y\right)\left(i\tilde{k}+X-Y\right)}. \tag{14}$$

We can similarly obtain the transparency ($S_{33}=1$) condition is

$$C_Y=\mp 1, X\mp Y=0. \tag{15}$$

Moreover, $X+Y=0$ yields T1: $\mu=-\frac{1}{2}(\lambda-3)(\lambda+1)$, while $X-Y=0$ yields T2: $\mu=\frac{3}{2}(\lambda+1)$.

We now explore the blockade ($S_{jj}=0$) condition for the incident wave $L_n^{(j)}$. In the denominator of Eq. (12), $X\pm Y$ can be transformed into

$$X+Y=\frac{(\lambda+1)(2\mu-3\lambda-3)}{\mu-2\lambda-2}, \tag{16}$$

$$X-Y=\frac{2\mu+\lambda^2-2\lambda-3}{\mu-2}. \tag{17}$$

The blockade can only occur at B1: $\mu=2\lambda+2=0$ (making $X+Y=\infty$), or B2: $\mu=2$ (making $X-Y=\infty$), when the numerator of S_{11} and S_{33} should be kept limited. In detail, if the blockade point is $\mu-2\lambda-2$ ($\mu=2$), to make $S_{11}=0$, there should be $\lim_{\mu\to 2\lambda+2\,(\mu\to 2)}(X+YC_Y)=Constant$, which yields $C_Y=-1$ ($C_Y=1$). Similarly, if the blockade point is $\mu-2\lambda-2$ ($\mu=2$), to make $S_{33}=0$ [see Eq. (14)], there should be $\lim_{\mu\to 2\lambda+2\,(\mu\to 2)}(X-YC_Y)=Constant$, which yields $C_Y=1$ ($C_Y=-1$). Note that when $C_Y=\pm 1$, the transmission coefficients are reduced to an analog form of the spinless case.?

Now we summarize the results. When $C_Y=-1$, we achieve $S_{11}=1$ and $S_{11}=0$ respectively at

$$\text{T1: } \mu=-\frac{1}{2}(\lambda-3)(\lambda+1) \text{ and B1: } \mu=2\lambda+2=0, \tag{18}$$

but achieve $S_{33}=1$ and $S_{33}=0$ respectively at

$$\text{T2: } \mu=\frac{3}{2}(\lambda+1) \text{ and B2: } \mu=2. \tag{19}$$

By comparison, when $C_Y=1$, we achieve $S_{11}=1$ and $S_{11}=0$ respectively at T2 and B2, but achieve $S_{33}=1$ and $S_{33}=0$ respectively at T1 and B1. Since we have assumed $b\in[0,\pi]$, then the condition $C_Y=\mp 1$ yields $b=\frac{\pi}{2}$, and $\varepsilon-a=\mp\frac{\pi}{2}$. Here, $b=\frac{\pi}{2}$ leads to $\mathbf{s}_{\pm,n}=\pm 2[\sin a\cos(2n\alpha)\mathbf{e}_x+\cos a\mathbf{e}_y-\sin a\sin(2n\alpha)\mathbf{e}_z]$, meaning $\mathbf{s}_{\pm,0}$ orients within the xoy plane [note that $\mathbf{s}_{\pm,n}$ represent the spin orientation of $l_n^{(1)}$ and $l_n^{(3)}$]. Furthermore, $\varepsilon-a=\mp\frac{\pi}{2}$ leads to $\mathbf{s}_{\varepsilon,n}=\pm\frac{2g}{\gamma}\kappa^{2|n|}[\sin a\cos(2n\alpha)\mathbf{e}_x+\cos a\mathbf{e}_y-\sin a\sin(2n\alpha)\mathbf{e}_z]=\frac{g}{\gamma}\kappa^{2|n|}\mathbf{s}_{\pm,n}$, meaning that the strong localized mode d_n should orient identical to $l_n^{(1)}$ or $l_n^{(3)}$. Here, T1, T2, B1 and B2 determine the energy ω at the transparency or blockade points. Moreover, $C_Y=\mp 1$ leads to $S_{31}=S_{13}=0$, signifying no conversion between $l_n^{(1)}$ and $l_n^{(3)}$ in the output fields.

Spin conversion

We now focus on the spin conversion. Our mission is to find the maximum conversion efficiencies S_{31} and S_{13}. Since $|S_{31}|=|S_{13}|$, we only need to investigate

$$S_{31}=\frac{i\tilde{k}(iY)\left(ie^{ib}\sin a\sin\varepsilon-C_\varepsilon\cos\varepsilon\right)}{(i\tilde{k}+X)^2-Y^2} \tag{20}$$

where $C_\varepsilon=\cos^2(a/2)+e^{i2b}\sin^2(a/2)$.

We define $M=ie^{ib}\sin a\sin\varepsilon-C_\varepsilon\cos\varepsilon$, which is independent of $\tilde{k}=2g^{-1}\sin k$. To examine the maximum value of M, we have

$$|M|^2=\frac{1}{2}\left(1+\sin^2 a\cos^2 b\right)+\frac{1}{2}(\cos^2 a+\sin^2 a\sin^2 b)\cos\left(2\varepsilon-2\theta'\right). \tag{21}$$

Here, θ' is determined by the relation $\tan 2\theta' = \sin 2a \sin b / \left(\cos^2 a - \sin^2 a \sin^2 b\right)$, which can be simplified into $\tan\theta' = \tan a \sin b$. First, it is obvious that $|M|^2 \geq \sin^2 a \cos^2 b$, where the lower bound is achieved when $\varepsilon = \theta' \pm \frac{\pi}{2}$. In particular, if $b = \frac{\pi}{2}$, the lower bound becomes zero with $\varepsilon = a \pm \frac{\pi}{2}$, which is the very condition for transparency and blockade. Similarly, we also have $|M|^2 \leq 1$, where the upper bound is achieved when $\varepsilon = \theta'$ or $\varepsilon = \theta' + \pi$, i.e., $\tan\varepsilon = \tan a \sin b$. This condition makes $C_Y = 0$, meaning the transport is spin-reciprocal, e.g., $S_{11} = S_{33}$.

Suppose $|M| = 1$ is achieved by setting $\tan\varepsilon = \tan a \sin b$, we now continue to maximize $|S_{31}|$ via examining $\tilde{k}$ (see Fig. 1). In this manner, we can easily obtain

$$|S_{31}|^2 = \frac{\tilde{k}^2 Y^2}{[\tilde{k}^2 + (X+Y)^2][\tilde{k}^2 + (X-Y)^2]} \leq \frac{1}{4}, \tag{22}$$

where the "=" sign is achieved at $\tilde{k}^2 = Y^2 - X^2$, i.e.,

$$4 - \omega^2 = g^2(Y^2 - X^2), \tag{23}$$

and the maximum $|S_{31}|$ is $\frac{1}{2}$. We can easily verify that the condition $\tilde{k}^2 = Y^2 - X^2$ and $\tan\varepsilon = \tan a \sin b$ also leads to $S_{11} = S_{33} = \frac{1}{2}$.

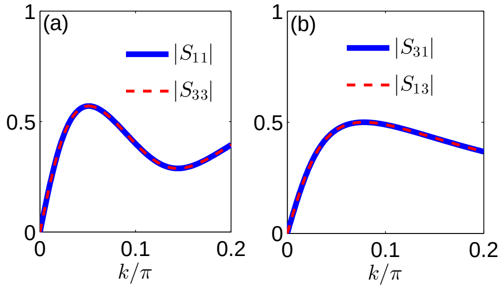

Figure 1. (color online). (a) Modulis of transmission coefficients S_{11} and S_{33}, and conversion efficiencies S_{31} and S_{13} for spin-reciprocal transport ($|S_{11}| = |S_{33}|$) at localization grade $g = 0.5$ and miscibility parameter $\lambda = 1$. Besides, we specify $\varepsilon = \arctan(\tan a \sin b)$, which favors the maximization of S_{31} and S_{13}.

References
